# *Primula xingyiensis* (Primulaceae), a new species in *Primula* sect. *Carolinella* from Guizhou, China

**DOI:** 10.3897/phytokeys.276.195710

**Published:** 2026-06-05

**Authors:** Xiang-ting Shi, Chao-yi Deng, Yuan-xing Lang, Zeng-cai Liu, Zhi-kun Wu

**Affiliations:** 1 Department of Pharmacy, Guizhou University of Traditional Chinese Medicine, Guiyang, 550025, Guizhou, China Department of Pharmacy, Guizhou University of Traditional Chinese Medicine Guiyang China; 2 Agriculture and Forestry Institute of Southwestern Guizhou, Xingyi 562400, Guizhou, China Agriculture and Forestry Institute of Southwestern Guizhou Xingyi China; 3 Xingyi Municipal Bureau of Agriculture and Rural Affairs, Xingyi, 562400, Guizhou, China Xingyi Municipal Bureau of Agriculture and Rural Affairs Xingyi China; 4 Environmental Monitoring Station of Qianxinan Prefecture, Xingyi, 562400, Guizhou, China Environmental Monitoring Station of Qianxinan Prefecture Xingyi China

**Keywords:** Conservation status, diversity, Guizhou, nomenclature, taxonomy, xing yi bao chun

## Abstract

*Primula xingyiensis* Z.K.Wu & C.Y.Deng, a new species of Primulaceae from Guizhou, China, is described and illustrated. Morphological evidence places *P. xingyiensis* within *Primula* sect. *Carolinella*, a section characterised by plants completely efarinose but bearing pubescent or glandular hairs; leaf base cordate to rounded, rarely broadly cuneate, with distinct petioles; calyx campanulate or narrowly campanulate, longer than its diameter, split to 1/3–1/2 of its length; and capsule oblong, dehiscing nearly calyptrate at maturity. The new species is distinguished by its gracile habit, shortened stem, small leaves with long villous hairs on both surfaces, long-styled homostylous flowers, and a corolla that is 7–8 times as long as the calyx. Information on the distribution, morphological comparisons with closely related species, and the conservation status of the new species is also provided, along with a key to the known species of *Primula* sect. *Carolinella*.

## Introduction

*Primula* L., the largest genus of the Primulaceae (Hu and Kelso 1996; Richards 2002), comprises approximately 549 species worldwide (POWO 2026) and serves as a valuable model for investigating the genetics and evolution of distyly (Darwin 1877; Mast et al. 2006; de Vos et al. 2013; Wang et al. 2021). The genus is primarily distributed in temperate and alpine regions of the Northern Hemisphere (Hu 1990a; Hu and Kelso 1996), with only a few species occurring in the Southern Hemisphere (Hu 1990a, 1994; Richards 2002). In China, approximately 351 species have been recorded (Liu 2025), which are classified into 24 sections and exhibit a high degree of endemism, with about 75% of the species being endemic to the country. Southwestern China, especially the Himalayan-Hengduan Mountains region, serves as both a centre of differentiation and a modern distribution centre for the genus, harbouring the vast majority of Chinese species, with concentrations in western Sichuan, eastern Xizang (Tibet), and northwestern Yunnan (Hu 1990a; Hu and Kelso 1996; Richards 2002; Yang et al. 2023).

*Primula* sect. *Carolinella* (Hemsl.) Pax is a small group within the genus *Primula*, comprising approximately 14 species worldwide. It is characterised by capsules that open by a lid (calyptrate) rather than by valves or by crumbling. Since the publication of the *Flora of China* (FOC), four newly described species belonging to this section have been published: *P. calyptrata* X.Gong & R.C.Fang (Gong and Fang 2003), *P. hunanensis* G.Hao, C.M.Hu & X.L.Yu (Yu et al. 2015), *P. undulifolia* G.Hao, C.M.Hu & Y.Xu (Xu et al. 2016), and *P. zhui* Y.H.Tan & B.Yang (Yang et al. 2017). Although the section is generally restricted to southeastern Yunnan, western Guangxi, southern Hunan and northern Guangdong, several of its members extend to the border regions of Vietnam and Thailand (Xu et al. 2016).

Guizhou Province in China, harbours remarkable biodiversity and is renowned worldwide for its karst topography and associated flora. It is recognised as one of the 36 global biodiversity hotspots (Feng et al. 2026). The heterogeneous habitats in this region have facilitated rapid diversification and speciation. Approximately 30 species of *Primula* have been documented in Guizhou, all of which occur in karst habitats (Yang et al. 2023). Among these, several endemic species are found, including *P. esquirolii* Petitm., *P. fangingensis* F.H.Chen & C.M.Hu, *P. kweichouensis* W.W.Smith, *P. lithophila* F.H.Chen & C.M.Hu, and *P. levicalyx* C.M.Hu & Z.R.Xu (Hu 1990a). In recent years, new species such as *P. chishuiensis* C.M.Hu, G. Hao & Y.Xu (Xu et al. 2022), *P. pingbaensis* NaZhang, X.Q.Jiang & Z.K.Wu (Zhang et al. 2023), and *P. xinjingensis* ShengH.Tang & F.W.Li (Tang et al. 2025) have been described from Guizhou, thereby enriching the species diversity of the genus *Primula* in the province.

During a botanical expedition to Xingyi, Guizhou Province, southwestern China, in December 2017, one of the authors discovered a distinctive species of *Primula* growing in karst mountains. The plant is gracile and dwarf, with small, suborbicular leaves that are purple on the abaxial surface, densely covered with long villous hairs on both surfaces, and have a cordate base. The scape is short; the flowers are long-styled and homostylous; the calyx is narrowly campanulate; and the corolla tube is 7–8 times as long as the calyx. From 2023 to 2026, we conducted multiple field investigations at the type locality and its surrounding areas, during December and March to April for four consecutive years, to confirm the floral and fruit characteristics of this species. We found that its capsule is calyptrate (i.e., dehiscing by a lid). The morphological characters of this species are stable and clearly distinguish it from all known *Primula* species. After consulting the relevant literature and examining herbarium specimens, we conclude that it belongs to *Primula* sect. *Carolinella* and represents an undescribed taxon new to science. Accordingly, we herein illustrate and describe it as a new species.

## Materials and methods

Type specimens and living material of the new species were collected from Xingyi, Guizhou, China. Morphological observations, measurements, and descriptions were carried out on randomly selected 15 living individuals. Comparative morphological analyses with closely related species were conducted by examining specimens from KUN and online herbaria of HUH (https://data.huh.harvard.edu/databases/specimen_search.php?mode=details&id=68450), as well as from relevant literature (Yamazaki 1988; Gong and Fang 2003). Key morphological characters of *P. xingyiensis* and its allied species within *Primula* sect. *Carolinella*, including *P. calyptrata*, were measured using a Vernier calliper on living plants from their type localities; the morphological characters of *P. intanoensis* T.Yamazaki were taken from the protologue (Yamazaki 1988; Hu 1990b). The conservation status of the new species was assessed following the guidelines of the IUCN Red List categories and criteria (IUCN Standards and Petitions Committee 2024). To facilitate the identification of *Primula* sect. *Carolinella*, a key to the species within this section, based on the primary morphological differences among these 15 species, was constructed.

## Taxonomic treatment

### Primula xingyiensis

Taxon classificationPlantaeEricalesPrimulaceae

Z.K.Wu & C.Y.Deng sp. nov.

BAC42E46-7ABD-5CB6-800E-7F39228ABD55

urn:lsid:ipni.org:names:77381046-1

Figs 1, 2, 3

#### Diagnosis.

**Figure 1.**
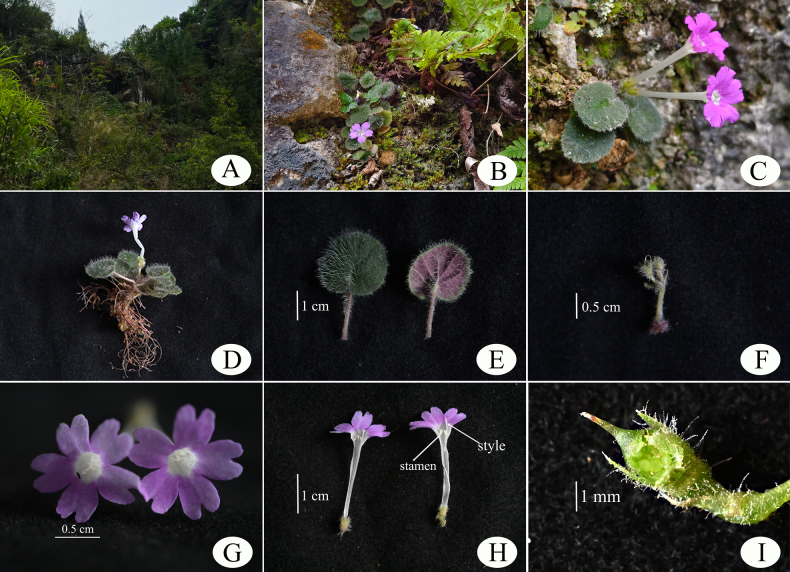
*Primula xingyiensis* sp. nov. **A, B**. Habitat; **C, D**. Habit during flowering; **E**. Leaves, the left: adaxial surface, the right: abaxial surface; **F**. Scape; **G**. Frontal view of flower; **H**. Dissected corolla showing anthers and stigmas; **I**. Capsule. Photographed by Zhikun Wu.

**Figure 2.**
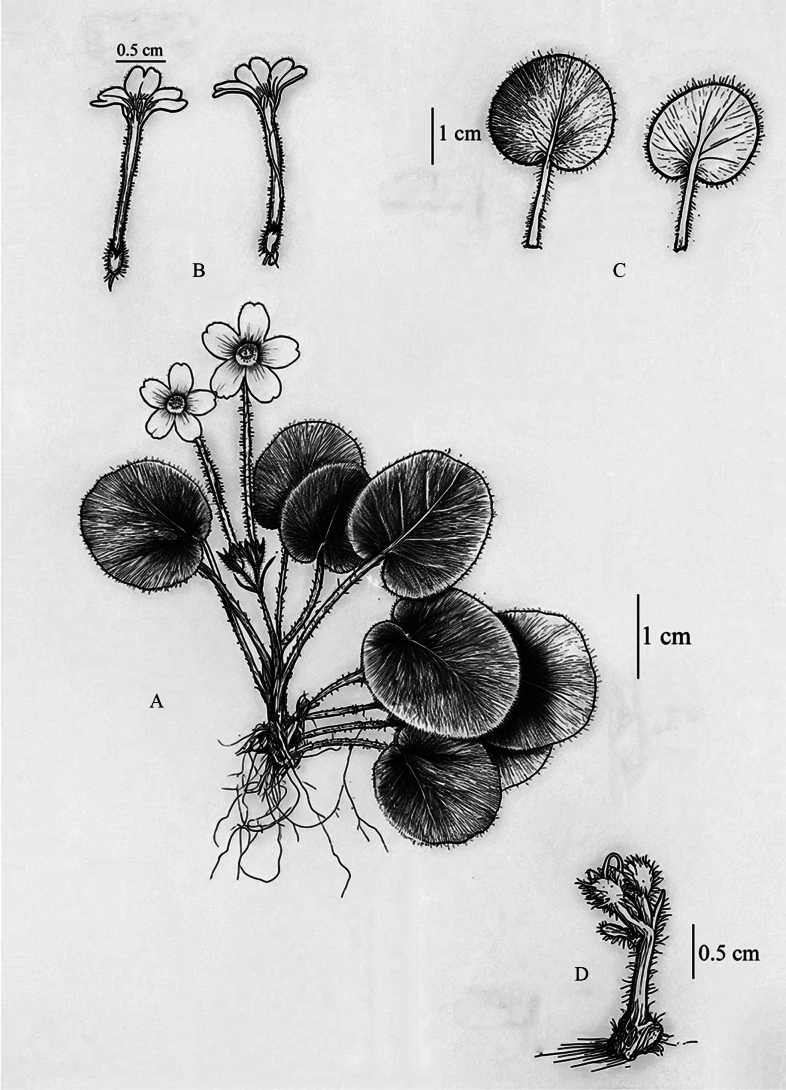
*Primula xingyiensis* sp. nov. **A**. Flowering habit; **B**. Dissected corolla; **C**. Leaves, the left: adaxial surface, the right: abaxial surface; **D**. Scape. Drawn by Ms. Xiangting Shi.

**Figure 3.**
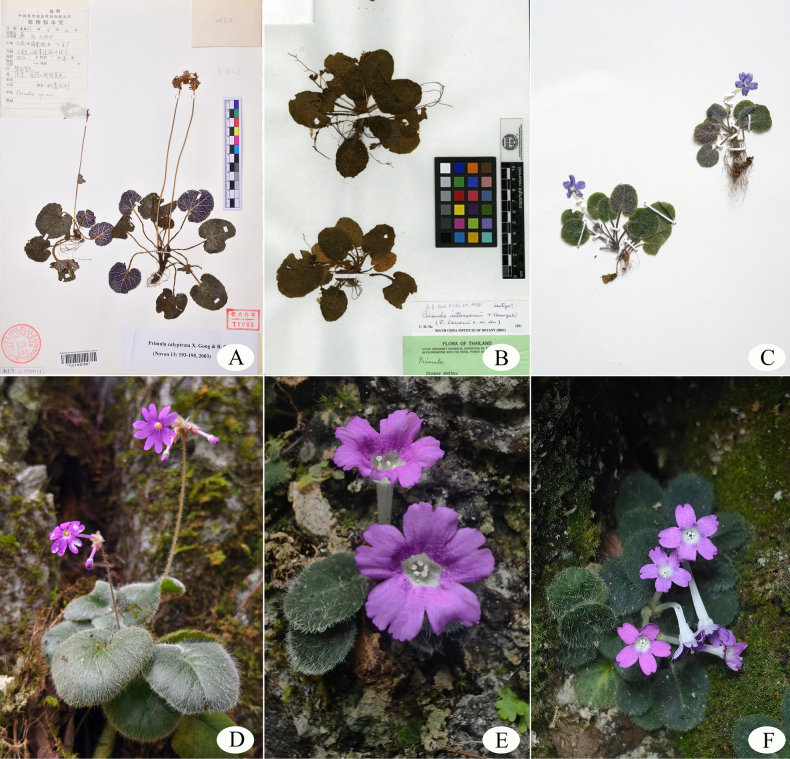
Two morphologically similar taxa and *Primula xingyiensis*. **A**. Holotype of *P. calyptrata* (X Gong 02815, KUN!); **B**. Isotype of *P. intanoensis* (M. Tagawa, K. Iwatsuki & N. Fukuoka 2863, HUH!); **C**. Holotype of *P. xingyiensis* (ZKWU2025210, KUN!); **D**. *P. calyptrata*; **E, F**. *P. xingyiensis*; **D–F**. Photographed by Zhikun Wu.

The new species is most similar to *P. intanoensis* and *P. calyptrata*, which are also long-styled homostylous species within this section, all sharing cordate leaves with long petioles and pubescent indumentum, as well as a campanulate or narrowly campanulate calyx. The new species differs from the latter two by its dwarf and gracile habit, small leaves only up to 1.7 cm wide, shortened scape measuring only 1–2 cm, shorter calyx 2–3 mm, and a long corolla tube that is 7–8 times as long as the calyx (Figs 1, 2, 3). The main morphological distinctions among *P. intanoensis*, *P. calyptrata* and *P. xingyiensis* are summarised in Table 1.

**Table 1.** Morphological comparison of *P. intanoensis*, *P. calyptrata* and *P. xingyiensis*.

Character	*P. intanoensis*	*P. calyptrata*	*P. xingyiensis*
Leaf blade size (cm)	3–5 × 2.5–4	2.8–4 × 2.6–3.8	1.1–1.7 × 1.1–1.7
Leaf abaxial colour	Green	Purple	Purplish-red
Calyx	Narrowly campanulate, 4–5 mm, lobes triangular	Campanulate, 5–6 mm long, lobes ovate-triangular	Narrowly campanulate, 2–3 mm long, lobes narrowly triangular
Corolla tube length (mm)	6–9 mm, 1–1.5 times the length of the calyx	ca. 17 mm, 3 times the length of the calyx	15–23 mm, 7–8 times the length of the calyx
Corolla colour	White	Purple	Pink with white tube
Scape height (cm)	Ca. 5	12–18	1–2

#### Type.

**China**. Guizhou: Xingyi city, Zhengtun town, Fengjiatian village. 25.0900, 105.0944, 1231 m alt., 12 November 2025 (fl.), *Zhikun Wu, ZKWU2025210* (holotype: KUN!; Isotype:KUN!).

#### Description.

Perennial herb, low-growing, delicate, entire plant efarinose; aerial parts pubescent. ***Rhizome*** short, fleshy, 1–1.5 cm long, 1–3 mm thick, sparsely branched, bearing a few fibrous roots. ***Leaves*** arranged in a basal rosette at the apex of the rhizome; petiole 1.5–3.5 cm long, densely villous, slightly expanded at base, purplish-red; leaf blade suborbicular, 1.1–1.7 cm long, 1.1–1.7 cm wide, apex rounded, base cordate, margin entire, both surfaces densely villous and white glandular dots; lateral veins 2–3 pairs, inconspicuous adaxially, abaxially purplish-red and prominent, sparsely villous along veins (midrib densely villous, lateral veins sparsely villous). ***Scape*** short, 1–2 cm long, elongating to 3 cm at fruiting; inflorescence an umbel, single-tiered, 2–6-flowered; bracts lanceolate, 2–3 mm long, externally villous; pedicel subequal to bracts in length, densely pubescent. ***Calyx*** 2–3 mm long, narrowly campanulate, villous, divided 1/3–1/2 its length; lobes narrowly triangular, apex acute. ***Flowers*** long-styled homostylous; corolla pink with a white tube, salverform; corolla tube pubescent outside, 1.5–2.3 cm long, 7–8 times as long as calyx; limb 1.1–1.6 cm in diameter; throat without an annulus, short-pubescent; corolla lobes oblong, apex deeply emarginate. ***Anthers*** positioned at the throat of corolla tube; style slightly exserted from the tube, 2–2.5 cm long. ***Capsule*** small, ca. 2 mm in diameter, dehiscing by circumscissile dehiscence. ***Seeds*** ca. 0.3 mm in diameter, surface smooth.

#### Distribution and ecology.

**Map 1.**
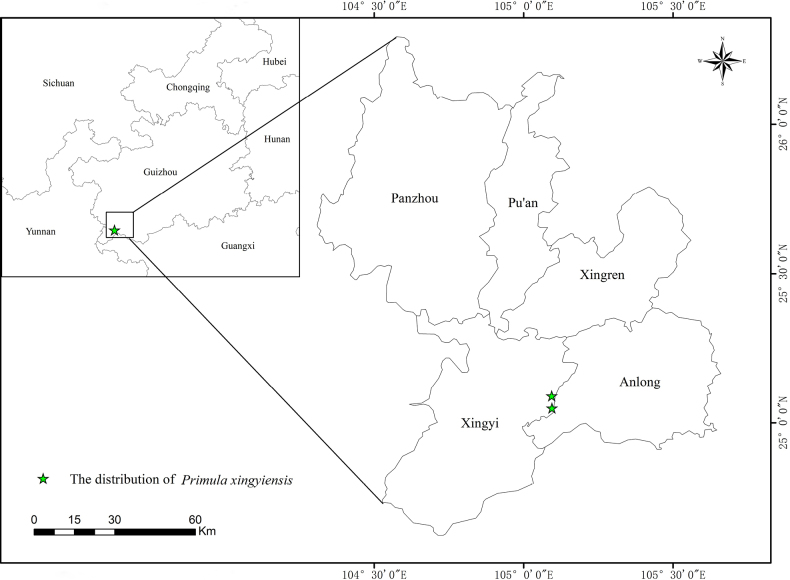
Location of the distribution of *Primula xingyiensis* in Xingyi, Guizhou.

Based on our field surveys in Xingyi and nearby karst areas of southwestern Guizhou, the new species *P. xingyiensis* is currently known only from two populations near the type locality, Xingyi, Guizhou, China. The species grows in moist crevices on shaded cliffs in karst mountains at elevations between 1200 and 1300 meters. (Fig. 1 A–C; Fig. 3 E, F; Map 1).

#### Phenology.

Flowering occurs from December to January of the following year, and fruiting occurs from February to April.

#### Etymology.

The specific epithet of the new species is taken from the Chinese Pinyin "Xingyi", the name of the county in southwestern Guizhou, China, where the type specimen was collected (Map 1).

#### Vernacular name.

Chinese mandarin: xing yi bao chun (兴义报春).

#### Provisional conservation status.

Critically Endangered (CR B2ab (i, ii, iii)). This new species exhibits a highly restricted distribution range. To date, it has been found only in two adjacent populations at the type locality, Zhengtun Town, Xingyi City. Nearly a decade of field observations indicate that the species grows in shady, moist crevices of cliffs within karst mountainous areas at elevations between 1200 and 1300 metres. The total number of mature individuals across the two populations is fewer than 200. The main threats include: (1) microhabitat drought resulting from climate change; and (2) human disturbance. Applying the IUCN Red List Categories and Criteria (Version 16.0, 2024), our assessment demonstrates that *P. xingyiensis* meets the threshold for Critically Endangered (CR) status under criterion B2ab (i, ii, iii), based on: extent of occurrence (AOO) < 500 km^2^ (B2), severely fragmented habitat (a), with continuing decline (b) in extent of occurrence (i), area of occupancy (ii) and area, extent and/or quality of habitat (iii).

#### Additional specimens examined.

**China** • Guizhou Province, Xingyi City, Zhengtun Town, Fengjiatian, the type location, 8 April 2026 (fr.), Chao-yi Deng and Yuan-xing Lang, 20260408001 (KUN!).

## Discussion

Subgenus *Carolinella*, characterised by its calyptrate capsule, comprises only section *Carolinella*. It is a relatively small group within the genus *Primula*, including 14 species (*P. partschiana* Pax, *P. carolinehenryae* S.O'Brien, *P. chapaensis* Gagnep., *P. kweichouensis*, *P. kwangtungensis* W.W.Smith, *P. cardioeides* W.W.Smith & H.R.Fletcher, *P. rugosa* N.P.Balakr., *P. levicalyx*, *P. intanoensis*, *P. wangii* F.H.Chen & C.M.Hu, *P. calyptrata*, *P. hunanensis*, *P. zhui*, and *P. undulifolia*). This group is endemic to southwestern China (Hunan, Guangdong, Guizhou, Guangxi and Yunnan) and adjacent Thailand and Vietnam. Previous studies have shown that pollen morphology within sect. *Carolinella* is polymorphic, indicating the heterogeneity of this group, and that its polycolpate pollen is of multiple origins (Anderberg and El-Ghazaly 2000). Further molecular phylogenetic investigations on subgenera *Auganthus* and *Carolinella* have demonstrated that the calyptrate capsule dehiscence is polyphyletic, and that the species of sect. *Carolinella* does not form a monophyletic group, and they are dispersed into four clades embedded within subgen. *Auganthus*, suggests that the species of this section should be merged into subgen. *Auganthus* (Yan et al. 2010; Liu et al. 2015; Xu et al. 2016). Morphologically, apart from the calyptrate capsule, the characters of sect. *Carolinella*, such as long petioles, pubescent leaves, cordate leaf bases, and the absence of farina on the whole plant, are more consistent with subgen. *Auganthus*. This resemblance has historically led to some members (e.g., *P. kwangtungensis*, *P. kweichouensis*) being placed in sect. *Obconicolisteri* for a long period (Smith and Fletcher 1946). Therefore, sect. *Carolinella* is unlikely to be monophyletic. However, because no formal molecular-based infrageneric treatment of *Primula* has yet been published, no definitive conclusion has been reached on the final taxonomic disposition of this group. Consequently, the delimitation of sect. *Carolinella* still relies on traditional taxonomic concepts. This is the reason why we place *P. xingyiensis* in this section. A more robust delimitation and subdivision of this group will depend on future phylogenetic studies based on comprehensive sampling.

Morphologically, *P. xingyiensis* is most similar to *P. intanoensis* and *P. calyptrata*, which also possess long-styled homostylous flowers. It can be easily distinguished from these two species by its gracile and dwarf habit, shortened scape, short calyx, and corolla tube that is 7–8 times as long as the calyx.

### Key to the species of *Primula* sect. *Carolinella*

To facilitate the identification of the 15 species in *Primula* sect. *Carolinella*, a key is constructed as follows:

**Table:** 

1	Inflorescences umbellate, umbels 1 or 2 superimposed, 2- to 15-flowered; leaf blade pubescent	**2**
–	Inflorescences abbreviated or almost umbellate, (6)12- to 28-flowered; leaf blade glabrous or glabrescent	**11**
2	Leaf blade suborbicular or ovate-elliptic, base distinctly cordate, flowers homostylous	**3**
–	Leaf blade oblong, elliptic, broadly ovate or broadly elliptic, base broadly cuneate or cordate, flowers heterostylous	**5**
3	Scape 12–18 cm long	***P. calyptrata* X. Gong & R.C.Fang**
–	Scape indistinct or short, 1–5 cm long	**4**
4	Corolla white, tube 6–9 mm long, nearly as long as the calyx or 1.5 times its length, scape up to 5 cm tall	***P. intanoensis* T.Yamazaki**
–	Corolla pink, tube 2–2.3 cm long, 7–8 times as long as calyx, scape up to 3 cm tall	***P. xingyiensis* Z.K.Wu & C.Y.Deng**
5	Pedicel and calyx glabrous	**6**
–	Pedicel and calyx fulvous pilose	**7**
6	Leaf blade oblong to broadly ovate, base cordate or subrounded, both surfaces densely pilose; calyx 3.5–4 mm, parted to near middle; corolla tube ca. 8 mm long	***P. levicalyx* C.M.Hu & Z.R.Xu**
–	Leaf blade oblong to oblong-obovate, base broadly cuneate, sometimes obtuse to subrounded, adaxially glabrous, abaxially sparsely pilose along veins; calyx 5.5–6 mm, parted to 1/3 of its length; corolla tube 12–13 mm long	***P. hunanensis* G.Hao, C.M.Hu & X.L.Yu**
7	Leaf blade base cordate or subrounded	**8**
–	Leaf blade base cuneate or rounded	**10**
8	Leaf blade ovate to ovate-elliptic, 6.5–13.5 cm long, corolla tube 14–16 mm long	***P. zhui* Y.H.Tan & B.Yang**
–	Leaf blade broadly oblong to suborbicular, 2.5–6.5 cm long, corolla tube ca. 10 mm long	**9**
9	Calyx 4.5–7.5 mm long, narrowly campanulate, parted to middle, lobes lanceolate	***P. wangii* F.H.Chen & C.M.Hu**
–	Calyx 5–5.5 mm long, campanulate, parted to 1/3, lobes obovate	***P. undulifolia* G.Hao, C.M.Hu & X.L.Yu**
10	Calyx 3–4 mm long, parted to 1/2–2/3 of its length	***P. kweichouensis* W.W.Smith**
–	Calyx 6–7 mm long, parted to ca. 1/3 of its length	***P. kwangtungensis* W.W.Smith**
11	Leaf blade acute or acuminate at apex	**12**
–	Leaf blade obtuse or rounded at apex	**13**
12	Leaf blade base rounded or somewhat cuneate; calyx ca. 3 mm long, inconspicuously 5-veined	***P. carolinehenryae* S.O'Brien**
–	Leaf blade base cordate; calyx 7–10 (12) mm long, prominently 10-ribbed	***P. chapaensis* Gagnepain**
13	Leaf blade base broadly cuneate to subrounded	***P. rugosa* N.P.Balakrishnan**
–	Leaf blade base cordate	**14**
14	Leaf blade elliptic-ovate to broadly ovate, 4.5–6 × 3.5–5.5 cm; petiole 2–5 cm long	***P. cardioeides* W.W.Smith & H.R.Fletcher**
–	Leaf blade oblong to suborbicular or ovate-rounded, 7–18 × 5–15 cm; petiole 7–26 cm long	***P. partschiana* Pax**

## Supplementary Material

XML Treatment for Primula xingyiensis

## Additional information

### Artificial Intelligence (AI) use

The authors accept full responsibility for the content of the manuscript, including the disclosure of any use of AI.

No AI tools were used in the preparation of this manuscript.

### Author ORCIDs

X-t. Shi https://orcid.org/0009-0007-4830-6526

C-y. Deng https://orcid.org/0000-0001-5407-5292

Y-x. Lang https://orcid.org/0000-0001-8175-0599

Z-c. Liu https://orcid.org/0000-0001-9866-0354

Z-k. Wu https://orcid.org/0000-0002-4583-7782

### Data availability

All of the data that support the findings of this study are available in the main text.
